# Screening and identification of host proteins interacting with *Toxoplasma gondii* SAG2 by yeast two-hybrid assay

**DOI:** 10.1186/s13071-017-2387-y

**Published:** 2017-10-02

**Authors:** Meng-Yee Lai, Yee-Ling Lau

**Affiliations:** Department of Parasitology, Faculty of Medicine, University of Malaya, 50603 Kuala Lumpur, Malaysia

**Keywords:** Co-immunoprecipitation, Protein-protein interaction, *Saccharomyces cerevisiae*, *Toxoplasma gondii*, Yeast two-hybrid

## Abstract

**Background:**

The identification of receptors or binding partners of *Toxoplasma gondii* from humans is an essential activity. Many proteins involved in *T. gondii* invasion have been characterized, and their contribution for parasite entry has been proposed. However, their molecular interactions remain unclear.

**Results:**

Yeast two-hybrid (Y2H) experiment was used to identify the binding partners of surface antigens of *T. gondii* by using SAG2 as bait*.* Colony PCR was performed and positive clones were sent for sequencing to confirm their identity. The yeast plasmids for true positive clones were rescued by transformation into *E. coli* TOP 10F’ cells. The interplay between bait and prey was confirmed by β-galactosidase assay and co-immunoprecipitation experiment. We detected 20 clones interacting with SAG2 based on a series of the selection procedures. Following the autoactivation and toxicity tests, SAG2 was proven to be a suitable candidate as a bait. Thirteen clones were further examined by small scale Y2H experiment. The results indicated that a strong interaction existed between *Homo sapiens* zinc finger protein and SAG2, which could activate the expressions of the reporter genes in diploid yeast. Co-immunoprecipitation experiment result indicated the binding between this prey and SAG2 protein was significant (Mann-Whitney U-test: *Z* = -1.964, *P* = 0.05).

**Conclusions:**

*Homo sapiens* zinc finger protein was found to interact with SAG2. To improve the understanding of this prey protein’s function, advanced investigations need to be carried out.

## Background

*Toxoplasma gondii* is an opportunistic intracellular protozoan parasite that invades all nucleated cells in humans, reptiles, birds and other warm-blooded animals. *Toxoplasma gondii* infection is globally distributed and 25–30% of the world’s human population was predicted to be infected by *T. gondii*, placing *T. gondii* among the most successful human parasites [1–5]. Definitive hosts for this parasite are species of the family Felidae. Humans become infected after consuming undercooked water or raw meats containing oocysts and tissue cysts of *T. gondii*, through the placenta from the mother to the fetus, blood transfusion, and organ transplantation. Usually, people with weak immune response (especially AIDS patients), immunocompromised patients, and newborn infants may develop severe diseases, such as pneumonia, encephalitis, mental retardation, and some other life-threatening conditions [6]. However, infection in healthy people will self-resolve and asymptomatic [7].

The yeast two-hybrid (Y2H) system developed by Fields & Song [8] utilized yeast as a tool to identify possible protein-protein interactions. Y2H is an in vivo approach and can be used to confirm putative interactions and define novel interacting domains [9]. Several strains of yeasts can be used as the host, such as *Saccharomyces cerevisiae* and *Pichia pastoris* [8, 10]. The Y2H system was designed based on the properties of the yeast transcription activator protein, GAL4. GAL4 protein consists of two main fragments, a DNA-binding domain (DNA-BD) and a DNA-activation domain (DNA-AD). A bait protein is expressed as a fusion to the GAL4 DNA-BD domain when GAL4 gene is activated. Similarly, the prey proteins are expressed as a fusion to the GAL4 DNA-AD domain when GAL4 gene is transcribed. When both of the bait and prey proteins interact, the DNA-BD and DNA-AD domains will reconstitute again and form a complete transcription factor (TF). TF recognizes the upstream activating sequence (UAS) and bind to the promoter and activate the transcription of the reporter genes, including nutritional markers and antibiotic selectable markers [9].

The glycophosphatidylinositol (GPI)-anchored antigens are distributed all over the surface of the *T. gondii* [11]. These molecules have the main responsibility of promoting the adherence of the *T. gondii* to the membrane of the host cells during the invasion process. Indeed, these molecules may provide an imperative protection to the parasites in order to survive in the host cell environment [12]. Most of the GPI antigens are found on the surface of the tachyzoites and bradyzoites [13, 14]. Among the antigens, SAG1 and SAG2 are the most important surface antigens.

Several studies have shown that SAG2 members took part in host cell attachment and invasion at which their antibodies are able to inhibit the attachment of the parasite on host cells [15, 16]. Such surface antigens included SRS (SAG1-related sequence) family, consisted of SAG1-like sequence branch and SAG2-like sequence branch [17]. To date, several SAG2 proteins such as SAG2A, SAG2B, SAG2C, SAG2D, SAG2X and SAG2Y have been identified [15]. In this study, SAG2A (P22) was used as the target gene. To our knowledge, there is no specific published report regarding the finding of receptors or interacted binding proteins from human cell to *T. gondii* by using SAG2 gene as the target. Hence, we attempted to identify the human host cell proteins that interact with this SAG2 during the host cell invasion. The possible interacting proteins or partners of *T. gondii* are identified from the cDNA human library through an Y2H experiment. The finalized interacted proteins were further confirmed by co-immunoprecipitation (co-IP) assay. Notably, the function of each of the possible interacted proteins was presumptively discussed.

## Methods

### Yeast strains and parasite strain

Two yeast strains of *S. cerevisiae*, Y2HGold and Y187 were used in this study (Clontech, California, USA). The Y2HGold yeast (*MATa*, *trp1-901*, *leu2-3*, *112*, *ura3-52*, *his3-200*, *gal4Δ*, *gal80Δ*, *LYS2::GAL1*_*UAS*_ *-Gal1*_*TATA*_ *-His3*, *GAL2*_*UAS*_ *-Gal2*_*TATA*_ *-Ade2URA3::MEL1*_*UAS*_ *-Mel1*_*TATA*_ *AUR1-C MEL1*) was employed as the bait strain. Meanwhile, Y187 yeast strain (*MATα*, *ura3-52*, *his3-200*, *ade2-101*, *trp1-901*, *leu2-3*, *112, gal4Δ*, *gal80Δ*, *met-*, *URA3::GAL1*_*UAS*_ *-Gal1*_*TATA*_ *-LacZ, MEL1*) was employed as a prey strain. Y2HGold contains four reporter genes, *AUR1*, *HIS3, ADE2* and *MEL1*, coding for enzyme inositol phosphhoryl ceramide synthase, histidine, adenine and α-galactosidase, respectively. Y187 possesses two reporter genes, *MEL1* and *LacZ*, coding for α-galactosidase and β-galactosidase enzymes, respectively.

*Toxoplasma gondii* RH strain was used in this study and maintained in HS27 cell lines. The tachyzoites were harvested after one week and used for DNA extraction.

### PCR amplification of the SAG2 gene

Tachyzoite DNA was extracted and purified from the Blood and Tissue Extraction Kit (Qiagen, Hilden, Germany) after washing with 1× PBS buffer. The DNA fragment encoding SAG2 gene without intron was amplified by SAG2 primers (F: 5′-GA CCA TGG CGT CCA CCA CCG GAC GCCA-3′ and R: 5′-GC CTG CAG TTA CAC AAA CGT GATC-3′). The SAG2 primers contained *Nco*I and *Pst*I restriction sites (underlined). These primers were designed based on the published SAG2 sequence (GenBank: FJ825705.1). The restriction sites were incorporated into both sets of primers in order to facilitate cloning of the PCR fragments into the corresponding restriction site of the pGEMT vector (Promega, Winsconsin, USA). PCR was performed in a final volume of 25 μl containing 4 μl of template DNA, 1× of buffer, 0.4 μM of each primer, 200 μM of dNTP mix, 1.5 mM of MgCl_2_ and 1 U of *Taq* DNA polymerase (Promega). The cycling condition was consisted of denaturation at 95 °C for 6 min, followed by 35 cycles of 95 °C for 30 s, 53 °C for 30 s, 72 °C for 1 min and a final extension at 72 °C for 5 min. The size of the amplicon generated by the SAG2 primers was 483 bp.

### Construction of bait plasmid

Amplified DNA fragment of SAG2 was purified, ligated into pGEMT vector and transformed into *E. coli* TOP10F’. Following the colony PCR, the plasmids were extracted from the positive recombinant clones using the QIAprep Spin Miniprep kit (Qiagen, Hilden, Germany) and sent for sequencing. Recombinant pGEMT-SAG2 plasmid was digested by *Nco*I and *Pst*I restriction enzymes and cloned into the corresponding restriction sites in pGBKT7 yeast vector. The recombinant pGBKT7-SAG2 plasmid was then transformed into Y2HGold with the aid of Yeastmaker™ Yeast Transformation System 2 kit according to the manufacturer’s manual (Clontech, Winsconsin, USA). The culture was plated on SD/-Trp agar plates and incubated at 30 °C for 5 days. Following the colony PCR, the yeast plasmid with insert was extracted by using Easy Yeast Plasmid Extraction kit (Clontech) and sent for sequencing to validate their identity.

### Bait plasmid expression

A single colony of recombinant pGBKT7-SAG2 bait was inoculated into 5 ml SD/-Trp broth and incubated at 30 °C with shaking at 200 rpm. On the next day, the entire overnight culture was inoculated into 50 ml YPDA broth and incubated at 30 °C with shaking at 200 rpm until the OD reached 0.4–0.6. The culture was centrifuged and the total protein was extracted by using urea/SDS method [18, 19]. Extracted proteins were then analyzed by 12% SDS-PAGE gels and transferred onto PVDF membrane (BioRad Laboratories, California, USA). The membrane was blotted with c-Myc tag mouse monoclonal antibody (Invitrogen, Carlsbad, CA, USA) for 1 h and followed by incubation with horseradish peroxidase conjugated goat anti-mouse secondary antibody (Invitrogen). The blot was observed using the direct ECL chemiluminescent method (GE, Healthcare, Illinois, USA). Yeast strain Y2HGold transformed with pGBKT7 vector was employed as a control.

### Autoactivation and toxicity test

It is important to test the bait protein for autoactivation prior the Y2H screening. The pGBKT7-SAG2 and pGBKT7 empty vector were transformed into Y2HGold yeast strain. The cultures were plated on SD/-Trp, SD/-Trp/X-α-Gal and SD/-Trp/X-α-Gal/AbA agar plates and incubated at 30 °C for 5 days. The colour and the size of the colonies were observed. Only the bait that was not toxic and do not autoactivate the reporter genes in the absence of prey protein was used in the following Y2H experiment.

### Yeast two-hybrid system (Y2H)

Y2H system was performed between recombinant pGBKT7-SAG2 (bait) and human cDNA human library (prey) (Clontech, USA). Human cDNA library was pre-transformed into Y187 yeast by cloning into the pGADT7-RecAB vector. The mated culture was plated on a series of selective agar plates including DDO, DDO/X/A, QDO, and QDO/X/A plates. To eliminate the false positive results, positive control and negative control were included in this experiment. The mating between Y2HGold yeast transformed with pGBKT7-53 and Y187 yeast cells transformed with pGADT7-T were employed as positive controls. Meanwhile, the mating between Y2HGold yeast transformed with pGBKT7-Lam and Y187 yeast transformed with pGADT7-T were used as negative controls.

At the end of the experiment, colony PCR was performed by using ADLD-Insert Screening Amplimer set (Clontech, USA). The PCR cycling conditions were as described above.

### Rescuing prey plasmid

To rescue the true positive prey plasmids, a single blue colony from Y2H experiment was re-streaked on DDO/X plates for two times and incubated for 5 days at 30 °C. The plasmids were extracted and transformed into *E. coli* Top10F' cells. The plasmids were sent for sequencing following the colony PCR amplification.

### Confirmation of interacted protein by small scale mating

To further confirm the genuine positive interaction between SAG2 and the potential prey proteins, a small scale Y2H assay was carried out. Generally, Y187 cells transformed with each of the prey plasmids were mated with the respective Y2HGold containing the pGBKT7-SAG2 and Y2HGold (pGBKT7). The mated culture was plated on QDO/X/A agar plates. The same positive and negative controls as in the Y2H experiment were included.

### Analysis of positive clones by β-galactosidase activity assay

The genuine positive clones were further analyzed using Yeast β-galactosidase Assay Kit (Thermo Fisher Scientific, Massachusetts, USA) following the manufacturer’s protocol. A portion of a single colony from a double dropout (DDO) plate was suspended with Y-PER reagent in order to lyse the yeast cells. The wavelength of the mixture was measured at OD 600 nm and 250 μl of 2× β-galactosidase Assay Buffer was added. The reaction was incubated at 37 °C until a colour change was observed. To stop the reaction, 200 μl of β-galactosidase Assay Stop Solution was added. The cell debris was then removed by centrifugation and the supernatant was measured at OD 420 nm. The β-galactosidase activity was calculated based on the equation provided from Yeast β-Galactosidase Assay Kit manual. The positive controls used in this assay were pGBKT7-53 and pGADT7-T. The enzyme activity was measured three times and the average β-galactosidase activity was calculated.

### Chemiluminescent co-immunoprecipitation

Binding strength between SAG2 and the interacted prey protein was examined by the co-IP method. The experiment was implemented by using Matchmaker™ Chemiluminescent co-IP System (Clontech, USA) according to the manufacturer’s standard protocol. Following the sequencing analysis and β-galactosidase activity test, *H. sapiens* zinc finger (HZF) protein was employed as the prey protein for *T. gondii* SAG2 protein. Briefly, the *T. gondii* SAG2 fragment was amplified using the primers F: 5′-AA GGA TCC TCC ACC ACC GAG ACG CCA-3′ and R: 5′-GC GGA TCC TTA CAC AAA CGT GATC-3′. Meanwhile, by using healthy human DNA as a template, the HZF region was amplified using the primers F: 5′-GC GGA TCC ATG GCT CAA GAA ACT AAT CAC-3′ and R: 5′-GC GGA TCC TCA AAT CTT TTG GAT CTT TTC ACC AAC AAC TAC TGG-3′. To facilitate cloning, *Bam*HI restriction site sequence was included into the primer sequences (underlined above). PCR cycling conditions were as described above. The size of the PCR amplicon for SAG2 and HZF genes are 483 bp and 627 bp, respectively. PCR products were purified and ligated into pGEMT vector system. Recombinant pGEMT-SAG2 and pGEMT-HZF plasmids were digested with *Bam*HI and sub-cloned into the respective pACGFP-C1 and pProLabel vectors followed by transformation into *E. coli* TOP10F’. After sequencing analysis, 4 μg of each recombinant pAcGFP-SAG2 and pProLabel-HZF plasmids were co-transfected into HEK 293 (ATCC® CRL-1573™) mammalian cells by using Lipofectamine 2000 reagents (Invitrogen, Carlsbad, CA, USA). The cells were grown in 6-well plate in Dulbecco’s modified Eagle medium (DMEM) supplemented with 10% fetal bovine serum and 1% of each of the sodium pyruvate, L-glutamine and Pen-step solution. One day after transfection, green fluorescent light was observed under a fluorescent microscope for the successfully transformed plasmids. Cells were harvested, lysed and ready for co-IP analyses. The cell lysates were incubated with anti-AcGFP1 polyclonal antibody at 4 °C. Luminescent signal from interacted protein was detected by using ProLabel Detection Kit II (Clontech, USA) following the manufacturer’s standard protocol. The relative luminescence units (RLU) of the samples was measured by using the Multimode Reader & Hydroflex Microplate Washer (Tecan, Zurich, Switzerland). Luminescent activity for interacting group was compared with two experimental controls, pAcGFP1-SAG2 with empty pProLabel vector and pProLabel-HZF with pAcGFP1 empty vector. Negative controls comprising pAcGFP1-Lam and ProLabel-T vectors (provided with the kit) were also included. The interaction was measured three times and the average RLU was calculated. Statistical significance of the differences between experimental sample and experimental controls was assessed with Prism5 software (GraphPad) using a nonparametric test (Mann-Whitney U-test).

## Results

PCR amplification successfully amplified the fragment of SAG2 from *T. gondii* tachyzoite strain and the size generated was 483 bp. The fragment was then purified and ligated into pGEMT vector and subcloned into pGBKT7 yeast vector. The extracted plasmid was sent for sequencing and the BLAST result showed a 100% similarity with *T. gondii* SAG2 (GenBank: FJ825705.1).

**Fig. 1:**
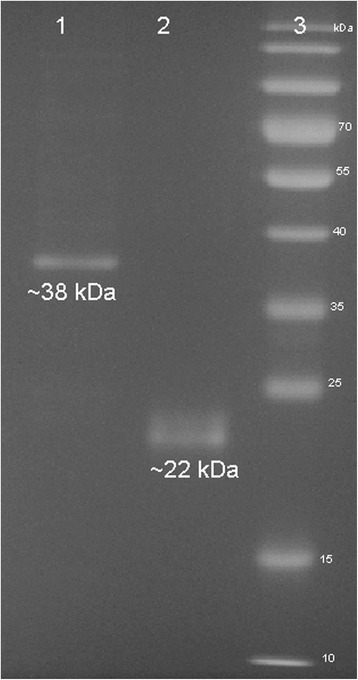
Western blotting detection of SAG2-pGBKT7 and pGBKT7 expression in Y2HGold cells. Lane 1: Expression of SAG2-PGBKT7 was detected at approximately 38 kDa size. Lane 2: Expression of empty pGBKT7 was detected at ~22 kDa. Lane 3: Protein ladder

Total proteins of the Y2HGold transformed with pGBKT7-SAG2 and pGBKT7 plasmids were analyzed by Western blotting and blotted with c-Myc-tag monoclonal antibody (Fig. 1). The molecular weight of pGBKT7-SAG2 and pGBKT7 was 38 kDa and 22 kDa, respectively. When introduced alone into yeast Y2HGold cells, pGBKT7 and pGBKT7-SAG2 alone did not autonomously activate the reporter gene. In addition, toxicity test for SAG2 showed that SAG2 bait was not toxic to yeast as the size of the colony was similar to that of Y2HGold transformed with the pGBKT7 empty vector. The autoactivation and toxicity test indicated that the constructs were suitable for use in Y2H screening.

**Fig. 2:**
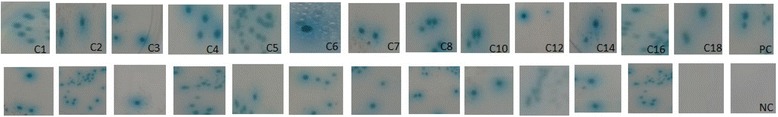
Confirmation of true positive clones by small-scale Y2H assay. Upper row: zygotes formed following the mating between pGBKT7-SAG2 plasmid and the respective prey 1 to prey 18 plasmids (C1-C18). Lower row: zygotes formed following the mating between pGBKT7 empty vector and the respective prey to prey 18 plasmids. PC: positive control, zygotes formed following the mating between Y2HGold transformed with pGBKT7-53 and Y187 transformed with pGADT7-T vector. NC: negative control, zygotes formed following the mating between Y2HGold transformed with pGBKT7 empty vector and Y187 transformed with pGADT7-T vector

From the Y2H experiment, approximately 4.6 × 10^6^ clones were screened resulting in 18 positive bait-prey interacting yeast clones (C1-C18). Sequence analysis of the plasmids indicated that these 18 plasmids, represent 13 *Homo sapiens* cDNA (Table 1). To confirm the specificity of the interaction, Y187 strain was transformed with each of the potential positive prey plasmid and mated with Y2HGold containing pGBKT7-SAG2 and pGBKT7 empty bait plasmid, respectively. Colonies were observed on QDO/X/A plates for all C1-C18 clones but no colonies were observed for C18 clones (Fig. 2). To eliminate the false positive result, controls were employed in this experiment. Blue colonies were observed on QDO/X/A plate for the positive control (Y187 yeast containing pGADT7-T mated with Y2HGold containing pGBKT7-53). On the other hand, no colonies were observed on QDO/X/A plate for the negative control (Y2HGold transformed with pGBKT7-Lam vector and pGADT7-T vector). In comparison with the results of the controls, one prey protein, C18, was identified to interact with SAG2 as no colonies were detected in QDO/X/A plate once mated with Y2HGold transformed with pGBKT7 empty plasmid. The clone was identified as *H. sapiens* zinc finger (HZF) protein (GenBank: NM_001242914.1) by DNA sequencing.

**Table 1:** BLAST result for interacting proteins between SAG2 and cDNA human library

Clone	Name	Functions	Accession number
1	*H. sapiens* phosphatase 4	Involved in Krebs Cycle	NR0035105
2	*H. sapiens* cathepsin B, mRNA	Cell division, PV and PVM formation	L22569
3	*H. sapien*s hydroxysteroid 17-beta dehydrogenase 6	Host cell invasion	NM_003725
4	*H. sapiens* Na+/Ca2+ exchanger, mRNA	Cell egression	AJ508602
5	*H. sapiens* keratin 222 (KRT222), mRNA	Host cell invasion	NM_152349
6	*H. sapiens* fasciculation and elongation protein zeta 2	Promote the growth of *T. gondii*	NM_005102
7	*H. sapiens* F-box protein 22, mRNA	Daughter cell division of *T. gondii*	BC008762
8	Human HS1 binding protein HAX-1, mRNA	Promotes the growth of *T. gondii*	U68566
9	Human HS1 binding protein HAX-1, mRNA	Promotes the growth of *T. gondii*	U68566
10	Human cAMP-dependent protein kinase	Promotes the cell division of *T. gondii*	NM_001028
11	*H. sapiens* Na+/Ca2+ exchanger isoform 4, mRNA	Cell egression	AJ508602
12	*H. sapiens* PTB domain adaptor protein CED-6, mRNA	Promotes the growth of *T. gondii*	AF200715
13	*H. sapiens* Na+/Ca2+ exchanger isoform 4, mRNA	Cell egression	AJ508602
14	*H. sapiens* cyclin C, mRNA	Cell differentiation	BC010135
15	*H. sapiens* cathepsin B, mRNA	Cell division, PV and PVM formation	L22569
16	*H. sapiens* endomucin, mRNA	Promotes the growth of *T. gondii*	AF205940
17	Human HS1 binding protein HAX-1, mRNA	Promotes the growth of *T. gondii*	U68566
18	*H. sapiens* zinc finger AN1-type	Differentiation of tachyzoite to bradyzoite	NM_001242914

**Fig. 3:**
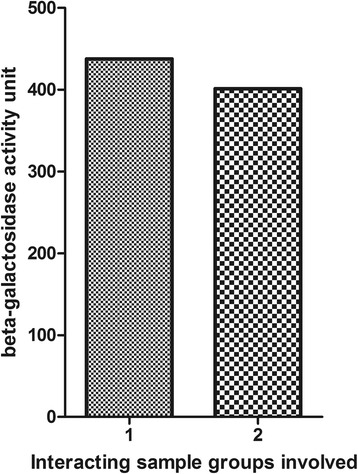
β-galactosidase activity of yeast transformed with pGBKT7-SAG2 and pGADT7-HZF in comparison to positive control. The β-galactosidase activity for the interaction between SAG2 and HZF was 437.7 units. Meanwhile, the β-galactosidase activity for the interaction between pGBKT7-53 and pGADT7-T (positive control) was 401.34 units. Interacting groups: 1: pGBKT7-SAG2 and pGADT7-HZF; 2: pGBKT7-53 and pGADT7-T; HZF: *H. sapiens* zinc finger protein

The strength of interaction between *T. gondii* SAG2 and HZF protein (a potential prey protein) was further measured by β-galactosidase activity assay. β-galactosidase activity for interaction between SAG2 and HZF was 437.7 units. Meanwhile, β-galactosidase activity for interaction between pGBKT7-53 and pGADT7-T (positive control) was 401.34 units. The solution turned yellow at 22 min for SAG2 and HZF and at 23 min for the positive control. β-galactosidase activity between SAG2 and HZF protein and the positive control are indicated in Fig. 3.

**Fig. 4:**
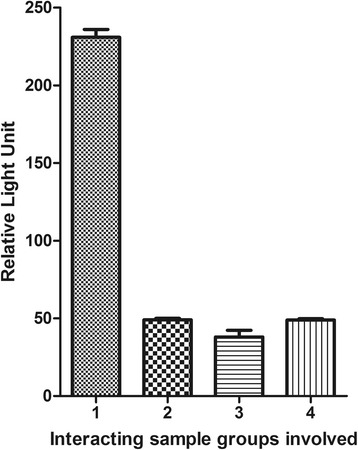
Chemiluminescent detection of the interaction between pAcGFP1-SAG2 and pProLabel-HZF by co-immunoprecipitation assay. RLU reading of pAcGFP1-SAG2 and pProLabel-HZF was found to be significantly different when compared to pAcGFP1-SAG2 and pProLabel protein (Mann-Whitney U-test, *Z* = -1.964, *P* = 0.05). Interacting groups 1: pAcGFP1-SAG2 + pProLabel-HZF; 2: pAcGFP1-Lam + pProLabel-T; 3: pAcGFP1-SAG2 + pProLabel; 4: pProLabel-HZF + pAcGFP1; HZF: *H. sapiens* zinc finger protein

Apart from this, the binding activity between HZF protein and *T. gondii* SAG2 was further confirmed by co-IP experiment. The interaction between HZF and SAG2 was found to be significant compared to experimental negative control (Fig. 4). Using the Mann-Whitney U-test, the RLU readings of pAcGFP1-SAG2 and pProLabel-HZF were found to be significantly different when compared with pAcGFP1-SAG2 and pProLabel proteins (*Z* = -1.964, *P* = 0.05). Meanwhile, in comparison with another experimental control, the RLU readings of pAcGFP1-SAG2 and pProLabel-HZF were found to be significantly different from pAcGFP1 and pProLabel-HZF protein (Mann-Whitney U-test: *Z* = -1.964, *P* = 0.05).

## Discussion

*Toxoplasma gondii* infects human populations worldwide, it is crucial to understand the interaction between the *T. gondii* antigen and the host cell proteins following invasion [20]. Protein-protein interactions play a pivotal role in cell division and different phases of cell cycle in all organisms [21]. *Toxoplasma gondii* SAG2 was used as the target gene throughout the present study to determine the potential binding partners from the human cDNA library.

We aimed to identify human proteins that were directly linked by physical associations with the *T. gondii* SAG2. SAG2 was found to interact with a series of host-cell proteins including functional enzymes, structural and functional organelle proteins. We succeeded to compile a list of the proteins that are involved in the attachment, invasion, penetration, PV and PVM formation, cell division, cell proliferation and cell egression (Table 1).

Although HZF is a host cytoplasmic protein, the results indicated that a strong interaction exists between HZF and SAG2. The band size for SAG2 was 38 kDa as SAG2 was expressed as a fusion to the Gal4 DNA-binding domain (Fig. 1). The cytoplasmic protein HZF may interact with SAG2 surface protein by using cell signaling molecules or hormones [22]. HZF proteins have been observed to interact with SAG2 to facilitate differentiation of *T. gondii* from tachyzoites to bradyzoites during the invasion process in the human body [23, 24]. During the asexual life-cycle of *T. gondii* in humans, the interconversion between the tachyzoite and the bradyzoite is crucial for survival and pathogenicity [23]. Zinc finger proteins have a diverse role in DNA recognition, RNA packaging, lipid binding, protein folding and manipulate the apoptosis pathway. Since zinc finger proteins contain a classical Cys_2_His_2_ motif, zinc finger proteins have a new important role involved in mechanisms of DNA binding and transcriptional regulation [24].

A single zinc finger structure was first reported 17 years ago by Lee et al. [25]. Wolfe et al. [26] elucidated that binding affinity for zinc finger structure was determined by the interaction between phosphate backbone and the adjacent zinc fingers. Also, side chain-base of zinc finger structure plays a pivotal role in determination of specificity binding [26]. Ultimately, Cys_2_His_2_ zinc finger proteins may play an important role in DNA binding due to their highly conserved linker sequence, TGEKP. This phenomenon had been proven by performing a single site mutagenesis. The result indicated that the binding affinity was reduced as much as 20-fold [27]. Apart from nucleic acid binding, several other functions of zinc finger proteins also had been reported. For example, zinc finger protein Ikaros is involved in lymphoid differentiation by connecting the two C-terminal Cys_2_His_2_ of zinc finger motifs [28].

To our knowledge, there are diverse motifs of zinc finger proteins and thus zinc finger proteins possess different functions. Those motifs are GATA-1 motif, β-ribbon motif as well as FYVE motif [24, 29].

As mentioned earlier, *T. gondii* surface SAG2 antigens played a pivotal role in host cell attachment and invasion [15, 16]. However, the mechanisms of interaction between SAG2 and HZF proteins need to be further studied. In order to understand further relevant interactions between SAG2 proteins and host cells, protein modelling may need to be performed. By observing the predicted three-dimensional structure of this SAG2 protein, its function may correlate with HZF protein. Protein modelling is a useful method to further investigate the interaction between two proteins. Junior et al. [30] reported that *T. gondii* SAG2A strongly interacts with both infected host innate and adaptive immune compartments. From their predicted protein structure modeling, SAG2A possessed an unfolded C-terminal end, which correlates the features of intrinsically unstructured proteins (IUP). Since IUP was able to interact with different molecules within the cells, IUP was associated with a variety of neurodegenerative diseases and viral virulence elements [30].

## Conclusions

HZF protein was found to interact with *T. gondii* SAG2. Nevertheless, more specific binding assays, such as localization studies and a pull-down assay can be performed to further confirm these interactions. To this end, a better understanding of the interplay between *T. gondii* and its hosts may prompt the development of new candidates for drug targets.

## Abbreviations

AbA: Antibiotic Aureobasidin A
AD: Activation domain
BD: Binding domain
CDK: Cyclin-dependent kinase
Co-IP: Co-immunoprecipitation
DDO: Double dropout with SD/−Leu/−Trp
DDO/X: SD/-Leu/-Trp supplemented with X-α-Gal
DDO/X/A: SD/-Leu/-Trp supplemented with X-α-Gal and Aureobasidin A
QDO: Quadruple dropout with SD/-Ade/-Trp/-Leu/-His
DMEM: Dulbecco’s modified Eagle medium
GPI: Glycophosphatidylinositol
HZF: *Homo sapiens* zinc finger
QDO/X/A: SD/-Ade/-Trp/-Leu/-His supplemented with X-α-Gal and Aureobasidin A
RLU: Relative light unit
SAG: Surface antigen
SD: Single dropout
Y2H: Yeast two-hybrid
YPD: Yeast medium with a blend of yeast extract, peptone and dextrose
YPDA: YPD with supplemented of adenine hemisulfate
